# Impact of diffused versus vasculature targeted DNA damage on the heart of mice depleted of telomeric factor Ft1

**DOI:** 10.1111/acel.14022

**Published:** 2023-11-13

**Authors:** Mattia La Torre, Eleonora Centofante, Carmine Nicoletti, Romina Burla, Alessandro Giampietro, Federica Cannistrà, Leonardo Schirone, Valentina Valenti, Sebastiano Sciarretta, Antonio Musarò, Isabella Saggio

**Affiliations:** ^1^ Department Biology and Biotechnologies “Charles Darwin” Sapienza University Rome Italy; ^2^ DAHFMO‐Unit of Histology and Medical Embryology Sapienza University Rome Italy; ^3^ Istituto Pasteur Fondazione Cenci Bolognetti Rome Italy; ^4^ CNR Institute of Molecular Biology and Pathology Rome Italy; ^5^ IRCCS Neuromed Pozzilli IS Italy; ^6^ Department of Cardiology Santa Maria Goretti Hospital Latina Italy; ^7^ Department Medical and Surgical Sciences and Biotechnologies Sapienza University Rome Italy; ^8^ School of Biological Sciences Nanyang Technological University Singapore Singapore; ^9^ NISB Institute of Structural Biology Nanyang Technological University Singapore Singapore

**Keywords:** cardiomyopathy, DNA damage, fibrosis, hypertrophy, telomeres

## Abstract

DNA damage is emerging as a driver of heart disease, although the cascade of events, its timing, and the cell types involved are yet to be fully clarified. In this context, the implication of cardiomyocytes has been highlighted, while that of vasculature smooth muscle cells has been implicated but not explored exhaustively. In our previous work we characterized a factor called Ft1 in mice and AKTIP in humans whose depletion generates telomere instability and DNA damage. Herein, we explored the effect of the reduction of Ft1 on the heart with the goal of comparatively defining the impact of DNA damage targeted to vasculature smooth muscle cells to that of diffuse damage. Using two newly generated mouse models, Ft1 constitutively knocked out (Ft1ko) mice, and mice in which we targeted the Ft1 depletion to the smooth muscle cells (Ft1sm22ko), it is shown that both genetic models display cardiac defects but with differences. Both Ft1ko and Ft1sm22ko mice display hypertrophy, fibrosis, and functional heart defects. Interestingly, Ft1sm22ko mice have early milder pathological traits that become manifest with age. Significantly, the defects of Ft1ko mice, including the alteration of the left ventricle and functional heart defects, are rescued by depletion of the DNA damage sensor p53. These results point to Ft1 deficiency as a driver of cardiac disease and show that Ft1 deficiency targeted to vasculature smooth muscle cells generates a pre‐pathological profile exacerbated by age.

## Graphical abstract

Ft1ko mice, with diffused reduction of the telomeric gene Ft1, and Ft1sm22ko mice, with vasculature targeted Ft1 depletion, display cardiac defects but with differences. Gross and molecular cardiac defects are more significant in Ft1ko mice and include left ventricle alterations. Both Ft1ko and Ft1sm22ko mice display acute inflammation, hypertrophy, and fibrosis. In Ft1sm22ko mice body weight defects and survival fragility become manifest with age.

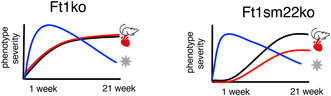

**Abbreviations**
Acta1: actin alpha 1
ADAM: a disintegrin and metalloprotease
AKTIP: AKT interacting protein
ANP: atrial natriuretic peptide
BNP: natriuretic peptide B
BP: blood pressure
BSA: bovine serum albumin
BW: body weight
Col‐: collagens
CSA: fiber cross‐sectional area
ERCC1: excision repair cross‐complementation group 1
ESCRT: endosomal sorting complexes required for transport
FISH: fluorescence in situ hybridization
Ft1: fused toes gene 1
Ft1F: Ft1 floxed
Ft1hz: constitutively knock out of Ft1 ‐ heterozygous mice
Ft1ko: constitutively knock out of Ft1 ‐ homozygous mice
Ft1kof: Ft1 knockout‐first mice
Ft1sm22ko: vasculature targeted depletion of Ft1
gDNA: genomic DNA
GLM: generalized linear model
HW: heart weight
IL‐: interleukins
LV: left ventricle
LV: lentivector
MAPK: mitogen‐activated protein kinase
MCP1: monocyte chemoattractant protein‐1
MMP: matrix metalloproteinase
mTOR: mechanistic target of rapamycin kinase
Myh6: myosin heavy chain 6
Myh7: myosin heavy chain 7
Ndufb7: NADH:ubiquinone oxidoreductase subunit B7
NFkB: nuclear factor kappa‐light‐chain‐enhancer of activated B cells
PBS: phosphate buffered saline
PINP: procollagen type I N‐terminal propeptide
Pla2g2a: phospholipase A2 group IIA
PPARGC1A: PPARG coactivator 1 alpha
Q‐PCR: quantitative PCR
RNAseq: RNA sequencing
SASP: senescence associated secretory phenotype
Slc39a8: solute carrier family 39 member 8
SPEN: spen family transcriptional repressor
Tgfβ: transforming growth factor beta
TL: tibia length
Tnc: tenascin C
TNFα: tumor necrosis factor alpha
Tnni3: troponin I3
Tnnt2: troponin T2
TSG101: tumor susceptibility gene 101
Ttn: titin
WT: wild type
XPG: xeroderma pigmentosum complementation group G

## 1 INTRODUCTION

Heart disease is a leading cause of death worldwide and refers to a range of conditions, including fibrosis, maladaptive hypertrophy, and left ventricle enlargement. Fibrosis refers to the excessive deposition of the extracellular matrix, particularly collagen, in heart tissue (Berk et al., 2007; McLellan et al., 2020). Post‐developmental hypertrophy occurs as an adaptive response to increased demands on the heart, and maladaptive hypertrophy occurs in response to pathological stimuli that is characterized by an abnormal increase in cardiomyocyte size and changes in gene expression (Nakamura & Sadoshima, 2018). Maladaptive hypertrophy can lead to impairment of cardiac function, stiffness, and increased risk of heart failure. Left ventricle structural changes occur in response to various conditions and are phenotypical traits of functional impairment (Nakamura & Sadoshima, 2018, Nishikawa, 2006 #69). Hypertrophy is defined by the activation of the MAPK, NFkB, AKT, and mTOR pathways (Samak et al., 2016). The modulation of Tgfβ and collagen, and of collagen metabolism, via PINP, MMPs, and ADAMs (Ding et al., 2020) characterizes the fibrotic phenotype.

An emerging driver of heart disease is DNA damage (Abdellatif et al., 2023). DNA can be damaged by oxidative stress, chronic inflammation, environmental toxins, telomeric attrition, or genetic mutations (Fyhrquist et al., 2013). The accumulation of DNA damage creates pathological cardiac scenarios for which it is relevant to define the single players, their interplay, and hierarchy. For example, it is yet to be clarified which heart cell types are determinant for DNA damage‐linked heart pathology. In this context, the implication of cardiomyocytes has been highlighted (Abdellatif et al., 2023; Puente et al., 2014), while that of vasculature smooth muscle cells has been implicated but not explored exhaustively (Gray et al., 2015; Wu et al., 2023).

Downstream to DNA damage, cell senescence impacts on vascular smooth muscle and endothelial cells, cardiomyocytes, and heart immune cells, and is associated with cardiovascular disease (Campisi & d'Adda di Fagagna, 2007). SASP (Senescence Associated Secretory Phenotype) defines senescent cells which over‐express p53/p21CIP1/WAF1, p16INK4a/Rb, senescence‐associated β‐galactosidase, IL‐1α, −6, and −8, and metalloproteases as MMP‐2 and ‐9. Accumulation of senescent vascular smooth muscle cells is detected in atherosclerotic plaques, while resident heart immune cells contribute to the paracrine inflammatory‐SASP phenotype of tissue. The release of cardiovascular burden obtained with senolytics further supports the negative role of senescent cells in heart dysfunction (Campisi & d'Adda di Fagagna, 2007; Mehdizadeh et al., 2022; Rodier et al., 2009).

AKTIP (Ft1 in mouse) is a human gene whose properties intercept genome integrity and telomere function. AKTIP (or Ft1) deficiency generates telomere instability and DNA damage (Burla et al., 2015, 2016; Cenci et al., 2015). AKTIP and Ft1 have sequence similarity with TSG101, a tumor susceptibility gene that functions in viral budding and cytokinesis. We produced evidence that AKTIP is associated with ESCRT, a macromolecular machinery acting on membrane dynamics and nuclear envelope integrity (Merigliano et al., 2021). In vivo, the reduction of the mouse counterpart of AKTIP, Ft1, induces premature aging of skin and bone and cancer predisposition (Burla et al., 2018; La Torre et al., 2018).

Herein, we explored the effect of the reduction of Ft1 on the heart with the goal of comparatively defining the impact of DNA damage targeted to vasculature smooth muscle cells to that of diffuse DNA damage. To address these points, we generated mice constitutively depleted in Ft1 (Ft1ko) and smooth muscle targeted depleted animals (Ft1sm22ko) to investigate the specific implication of these cells in pathomolecular cardiac events.

Our results suggest that Ft1 deficiency is a driver of cardiac disease. Our data also show that Ft1 deficiency targeted to smooth muscle cells is sufficient to generate a pre‐pathological profile early in life that is exacerbated with aging. Constitutive depletion of Ft1, which includes its reduction in cardiomyocytes and fibroblasts, is, on the other hand, necessary for the generation of manifest cardiac pathological defects at the early age and later in life. Finally, we show that these defects are rescued by depletion of the DNA damage responder p53.

## 2 RESULTS

### 2.1 Production and colony characterization of mice with constitutive and vascular smooth muscle targeted depletion of Ft1

Ft1 is a factor needed for telomere integrity whose depletion induces DNA damage (Burla et al., 2015, 2016, 2018; La Torre et al., 2018). We explored the link between heart failure and DNA damage in relation to Ft1 function and the implication of vasculature targeted DNA damage in heart pathology. Toward this end two different Ft1ko models were designed. In the first, we constitutively knocked out Ft1 (Ft1ko mice). In the second, Ft1sm22ko mice, we targeted the depletion of Ft1 using the sm22 promoter, a prototypical marker of vasculature smooth muscle cells in the heart (Moessler et al., 1996; Skelly et al., 2018). To rule out that the phenotype of ko animals would be conditioned by organ or age differences related to Ft1, we assessed Ft1 expression in WT mice. Q‐PCR on the heart, aorta, brain, kidney, liver, and spleen showed that Ft1 is expressed at similar levels in all organs (Figure S1A,B), and that the expression of Ft1 in mice at 1 and 21 weeks in each organ is comparable (Figure S1C).

To generate Ft1ko mice, we started from Ft1 ko‐first (Ft1kof) animals (La Torre et al., 2018). Ft1kof genetics is based on a cassette flanked by flippase recognition sites (Frt) that can be readily used to generate further genetically modified animals (Testa et al., 2004). Ft1kof mice were crossed with flippase expressing mice (flpo/Gt(ROSA)26Sorflpo) (Birling et al., 2012) to obtain Ft1 floxed (FtF) (Figure S1D,E). Using Ft1F derived mouse embryonic fibroblasts transduced with a lentiviral vector expressing Cre recombinase targeting the Ft1 cassette, accumulation of DNA damage on ɣH2AX foci was observed (Figure S1F,G), as well as an increase in telomere number (Figure S1H,I). These data are consistent with the function played by Ft1 (Burla et al., 2015, 2016). Indeed, we previously showed that the depletion of Ft1 (or of its human orthologue AKTIP) generates genome instability and activation of a DNA damage response along with telomere fragility defined by the presence of multiple telomere signals at chromosome ends (Burla et al., 2015, 2016). Therefore, the phenotype of Ft1F derived mouse embryonic fibroblasts prefigurates the suitability of our models to explore the impact of Ft1 depletion. Crossing Ft1F with constitutively Cre expressing mice (CMV‐cre/Tg(CMV‐cre)1Cgn) (Schwenk et al., 1995) (Figure S1D) we generated Ft1ko mice, as validated by genotyping, before and after Cre removal (Figure S1J). For the production of vasculature targeted Ft1 depletion, Ft1F mice were crossed with sm22‐cre mice (sm22‐cre/Tg(Tagln‐cre)1Zli) (Moessler et al., 1996) (Figure S1K). The genotypes of Ft1sm22ko mice were validated by PCR before and after Cre removal (Figure S1L).

**FIGURE 1:**
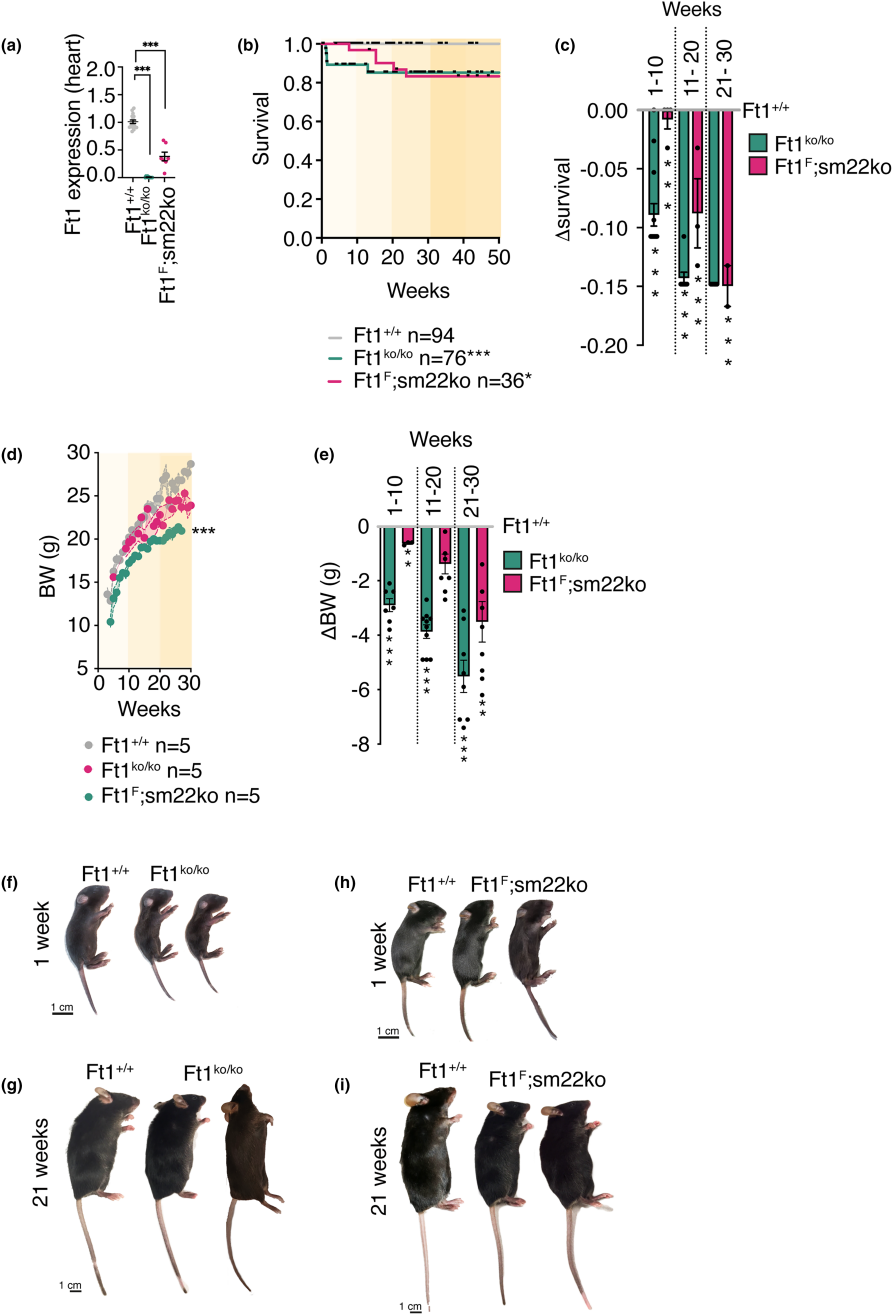
Survival and developmental alterations in Ft1ko and Ft1sm22ko mice. (a) Q‐PCR analysis of relative Ft1 expression in sex balanced populations of WT, Ft1ko, and Ft1sm22ko mice. (b) Kaplan–Meier survival curves of sex balanced populations of WT, Ft1ko, and Ft1sm22ko mouse colonies. (c) Delta survival proportion of sex balanced populations of Ft1ko and Ft1sm22ko versus WT mice. (d) Body weight (BW) curves of females of WT, Ft1ko, and Ft1sm22ko mice. (e) Delta BW of females of Ft1ko and Ft1sm22ko vs WT mice. (f–i) Representative images of WT and Ft1ko (f, g) and WT and Ft1sm22ko (h, i) of 1 and 21 week old mice, respectively. A minimum of five animals/genotype was used for analyses. Statistical analysis for survival curves were performed with log‐rank—Mantel–Cox test and shown in graphs with *p < 0.05, ***p < 0.001. Unless specified, statistical analysis for significant differences were performed with Student's test and shown in graphs by *p < 0.05, **p < 0.01, ***p < 0.001. Graphs show mean ± SEM.

As a preliminary step to colony characterization, we monitored Ft1 expression in the different models. Q‐PCR showed that Ft1 is at levels similar to WT in Ft1F (Figure S1M), was fully depleted in Ft1ko (Figure 1A), and was partially reduced in the heart of Ft1sm22ko animals; this latter trait is consistent with the fact that the heart contains both sm22 positive and sm22 negative cells (Figure 1A). To profile the overall impact of Ft1 depletion, we monitored the survival and body weight in the two mouse colonies over time. Constitutive depletion of Ft1 causes survival defects that are present as early as 1 week of age and remain significant over time, until old age (Figure 1B,C). The situation was different for mice with vascular depletion of Ft1, which display mild early survival defects that became more robust after 11 and 20 weeks (Figure 1B,C). The evaluation of body weight showed that there was a significant reduction in Ft1ko mice starting at neonatal age (Figure 1D–G). In Ft1sm22ko mice, we observed a modest reduction of body weight in neonatal mice compared to matched WT controls, but the defect was exacerbated in older mice (Figure 1D,E, H,I).

These data show that the depletion of Ft1 significantly affects mouse development and survival and that when the depletion is targeted to the vasculature the induced fragility has an increasing impact with age.

### 2.2 Constitutive and vasculature targeted Ft1 deficiency drive cardiac hypertrophy and fibrosis

**FIGURE 2:**
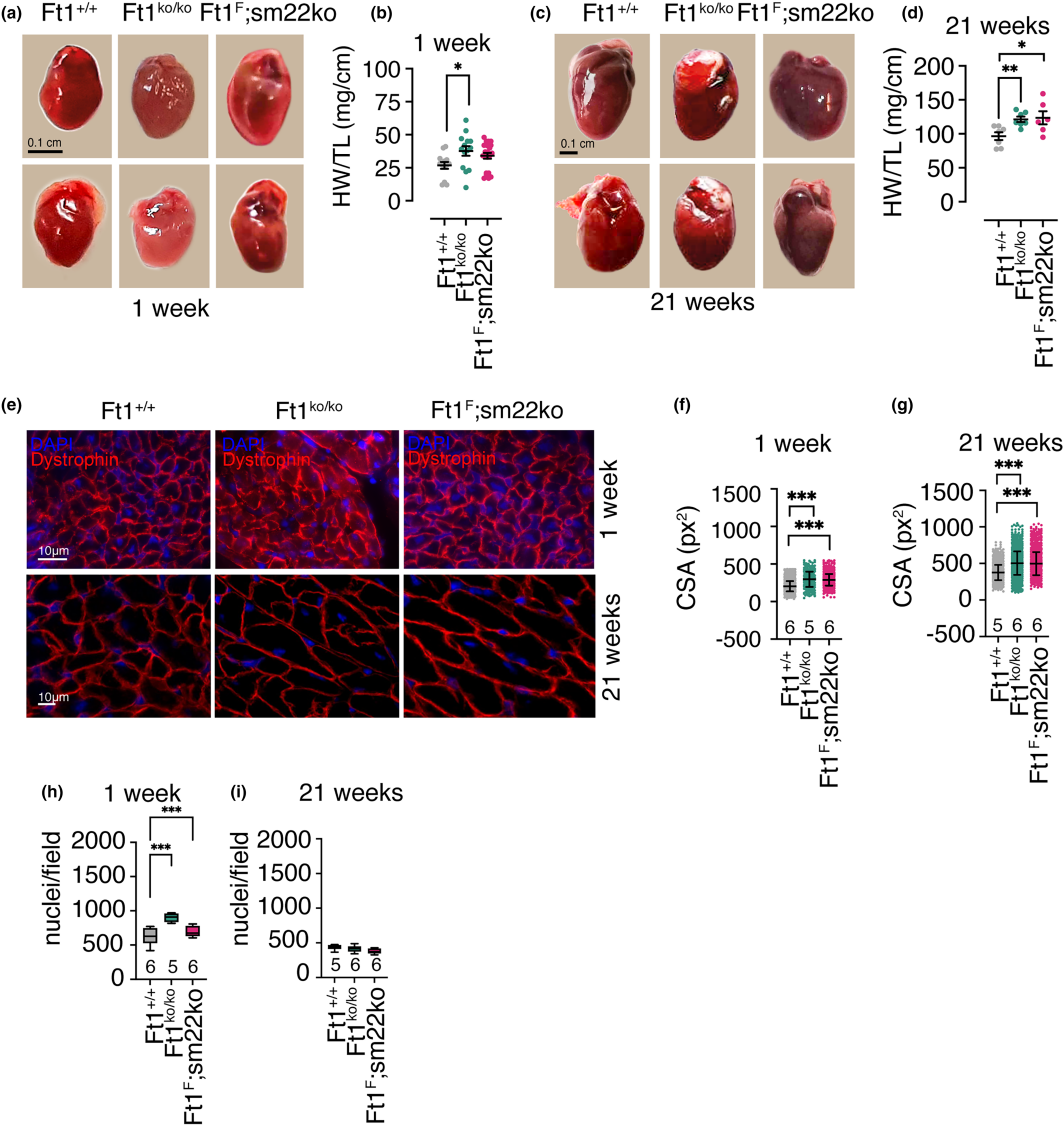
Gross heart alterations in Ft1ko and Ft1sm22ko mice. (a) Representative images of hearts from WT, Ft1ko, and Ft1sm22ko mice at 1 week. (b) Heart weight (HW) normalized to tibia length (TL) of WT, Ft1ko, and Ft1sm22ko mice at 1 week. (c) Representative images of hearts from WT, Ft1ko, and Ft1sm22ko mice at 21 weeks. (d) HW normalized to TL analysis of WT, Ft1ko, and Ft1sm22ko mice at 21 weeks. (e) Representative images of DAPI (blue) and dystrophin (red) stained transverse sections of the hearts from WT, Ft1ko, and Ft1sm22ko mice at 1 and 21 weeks. (f, g) Measure of cross‐sectional area (CSA) of WT, Ft1ko, and Ft1sm22ko mice at 1 and 21 weeks, respectively. Graphs show the mean ± SD. (h, i) Quantification of the number of nuclei per field in cardiac sections from WT, Ft1ko, and Ft1sm22ko mice at 1 and 21 weeks. A minimum of five animals/genotype was used for analyses. Analyses were performed on sex balanced populations. Statistical analysis for significant differences were performed with Student's test and shown in graphs by *p < 0.05, **p < 0.01, ***p < 0.001. Unless specified, graphs show the mean ± SEM.

For the study of cardiac defects, we focused on animal groups aged 1 and 21 weeks because data on survival and animal weight show clear‐cut differences between the two mouse models at these ages. Moreover, 1 week of age in rodents is the time limit for cardiomyocyte proliferation in the heart (Porrello et al., 2011; Puente et al., 2014), an aspect that is relevant for both response to damage of cardiac tissue and for analysis of Ft1, whose action is linked with DNA replication and cell division (Burla et al., 2015; Merigliano et al., 2021). Analysis of heart weight showed that it was greater in Ft1ko animals at both 1 and 21 weeks of age (Figure 2A–D). On the other hand, 1 week old mice with Ft1 depletion targeted to the vasculature did not display differences in heart weight (Figure 2A,B). However, these defects became significant in Ft1sm22ko mice at 21 weeks (Figure 2C,D). Measurement of the fiber cross‐sectional area (CSA) in cardiac transverse sections stained for dystrophin showed that Ft1 depletion caused a mild increase in CSA in both Ft1ko and Ft1sm22ko at 1 and 21 weeks (Figure 2E–G). Counting the number of nuclei in cardiac transversal sections, a robust increase in Ft1ko and Ft1sm22ko mice was seen at 1 week (Figure 2H). On the other hand, there was no difference in nuclear numbers compared to WT in the hearts obtained from Ft1ko and Ft1sm22ko mice at 21 weeks (Figure 2I). Together these data suggest that Ft1 depletion drives cardiac hypertrophy.

**FIGURE 3:**
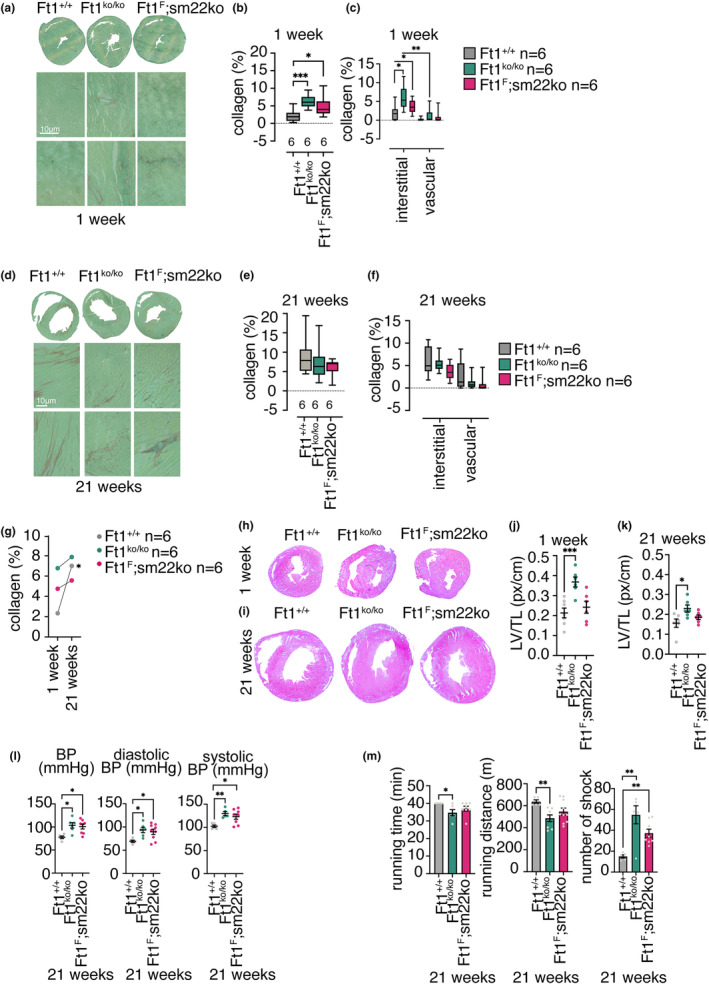
Fibrosis, left ventricle, and cardiac functions alterations of Ft1 depleted mice. (a) Representative images from fast green picrosirius stained transverse sections of the hearts from WT, Ft1ko, and Ft1sm22ko mice at 1 week. (b, c) Total and interstitial versus vascular collagen quantification from images in (a) in WT, Ft1ko, and Ft1sm22ko mice at 1 week. (d) Representative images from fast green picrosirius stained transverse sections of the hearts of WT, Ft1ko, and Ft1sm22ko mice at 21 weeks. (e, f) Total and interstitial vs vascular collagen quantification of hearts from WT, Ft1ko, and Ft1sm22ko mice at 21 weeks. (g) Quantification of collagen percentage at 1 and 21 weeks in WT, Ft1ko, and Ft1sm22ko mice. (h, i) Representative images from hematoxylin and eosin‐stained transverse sections of the hearts WT, Ft1ko, and Ft1sm22ko mice at 1 and 21 weeks. (j, k) Morphometric analyses of left ventricle (LV) walls normalized by TL of WT, Ft1ko, and Ft1sm22ko mice at 1 and 21 weeks. (l) Mean blood pressure (BP), diastolic and systolic BP measurements in WT, Ft1ko and Ft1sm22ko mice at 21 weeks. (m) Running time, distance and number of shocks observed in WT, Ft1ko, and Ft1sm22ko mice at 21 weeks during an exhaustion treadmill test. A minimum of five animals/genotype was used for analyses. Analyses were performed on sex balanced populations. Statistically analysis was performed with Student's test and shown in graphs by *p < 0.05, **p < 0.01, ***p < 0.001. Graphs show the mean ± SEM.

Pathological hypertrophy is found in association with fibrosis and abnormal collagen deposition (Berk et al., 2007; McLellan et al., 2020; Nakamura & Sadoshima, 2018). To monitor this latter trait in these mice, we stained myocardial sections with green picrosirius and quantified the collagen volume (Han et al., 2023). The constitutive depletion of Ft1 induced a significant accumulation of collagen, prevalently in interstitial space, rather than in proximity to the vasculature (Figure 3A–C). In Ft1sm22ko mice at 1 week, a similar but milder phenotype was observed (Figure 3A–C). In mice at 21 weeks, there was no further significant increase in collagen accumulation in either Ft1ko or Ft1sm22ko mice (Figure 3D–F). On the other hand, in WT animals collagen deposition increased with age (Figure 3G). Taken together this suggests that in Ft1 genetically modified animals the increase in collagen deposition in the heart occurs prematurely consequently to the heart damage induced by Ft1 depletion.

We next evaluated cardiac function in our genetic models by monitoring histological and functional parameters. Histologically, we observed an increase in left ventricle size in 1 week old Ft1ko mice (Figure 3H,J). A difference between Ft1ko and WT mice was also observed comparing mice at 21 weeks of age (Figure 3I,K). Functionally, monitoring mean, diastolic, and systolic blood pressure in 21 week old Ft1ko and Ft1sm22ko mice we observed a significant increase of blood pressure in mutant mice compared to WT (Figure 3L). Running performances, measured by the exhaustion treadmill test (O'Connell et al., 2003), revealed a decrease in running time and distance accompanied by an increase in the number of shocks in Ft1ko mice (Figure 3M). In Ft1sm22ko mice, a similar but milder phenotype was observed (Figure 3M).

These data suggest that both constitutive and vasculature targeted depletion of Ft1 drive hypertrophy, increased collagen deposition, and cardiac functional defects.

### 2.3 The constitutive and vasculature targeted depletion of Ft1 induce the alteration of the heart transcriptome

**FIGURE 4:**
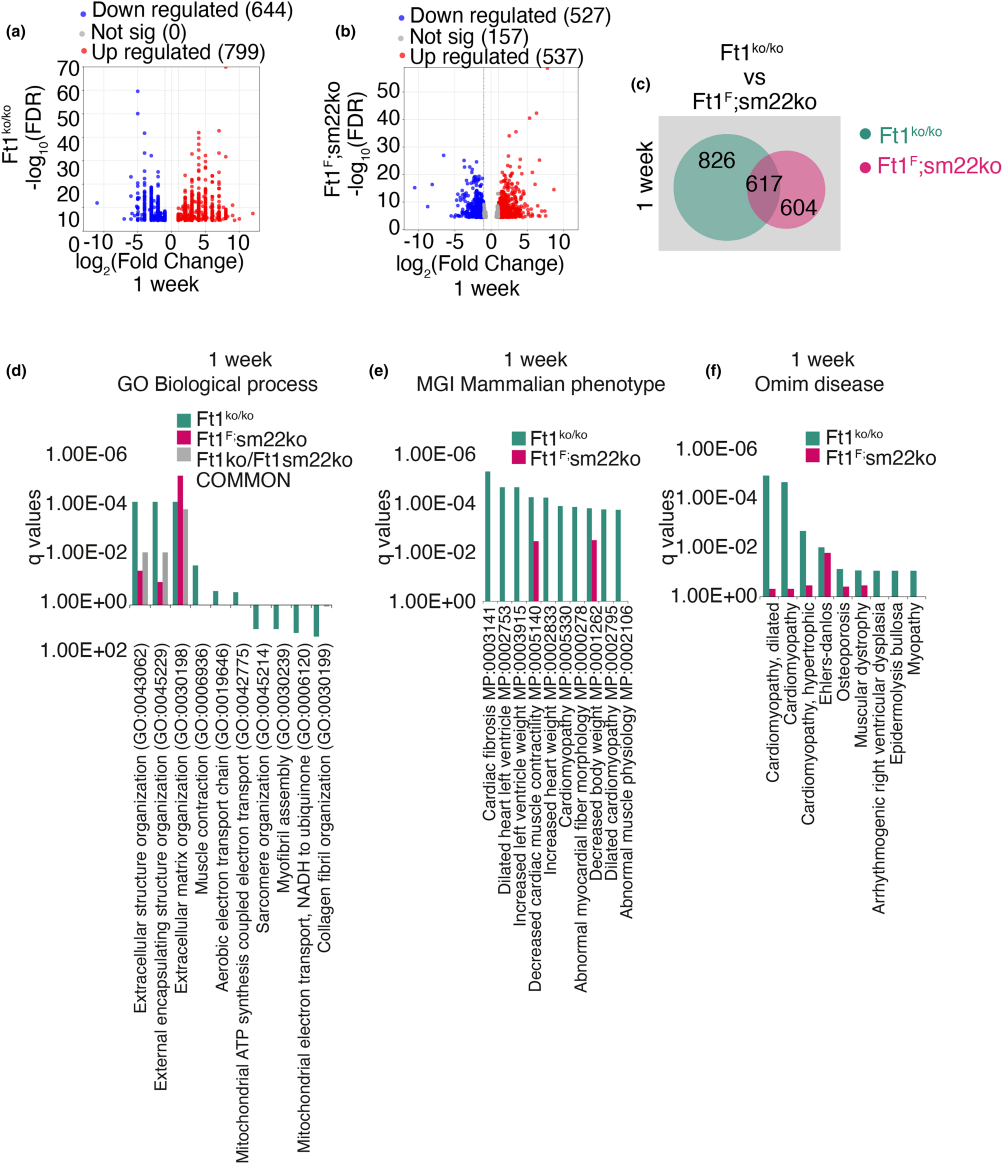
Heart transcriptome of Ft1 depleted 1 week old mice. (a, b) Volcano plot representing the total number of significantly modulated transcripts obtained by RNAseq analysis of hearts from males Ft1ko (*N* = 3) (a), Ft1sm22ko (*N* = 4) (b), at 1 week compared to matched WT (*N* = 3). (c) Venn diagram of differentially expressed transcripts from RNAseq analysis of Ft1ko (green) and Ft1sm22ko (pink) of 1 week old mice compared to matched WT. (d–f) Enrichr analysis for MGI Mammalian Phenotype Level 42,021, biological process, and Omim disease of significantly modulated transcripts from Ft1ko and Ft1sm22ko mice at 1 week. Significance was obtained with Bonferroni correction and FDR <0.05.

To complete the characterization of Ft1ko and Ft1sm22ko mice and interpret the heart phenotypes through a comprehensive molecular lens, we performed RNAseq of cardiac tissue from animals at 1 week of age. We focused on this age for 1 week is the time window for heart regeneration and active cell proliferation (Porrello et al., 2011; Puente et al., 2014). Transcript alignment of RNAseq samples on the region encompassing the deletion in the Ft1 gene shows, as expected, the absence of transcripts in Ft1ko mice and a reduction in Ft1sm22ko mice (Figure S2A–C). Overall, the analyses demonstrated that Ft1 depletion generated significant modulation of the cardiac transcriptome, with 1443 transcripts modulated in Ft1ko and 1064 in Ft1sm22ko mice (Figure 4A,B). In Ft1ko mice, 799 transcripts were upregulated and 644 were downregulated (Figure 4A). In 1 week old Ft1sm22ko mice, 527 transcripts were upregulated and 537 were downregulated (Figure 4B). An abundant number of transcripts is commonly regulated in Ft1ko and Ft1sm22ko mice (*n* = 617; Figure 4C). Interrogation of the gene ontology (GO) data sets indicated that the extracellular organization of the hearts in both Ft1ko and Ft1sm22ko mice was altered (Figure 4D), and that in Ft1ko there was alteration of transcripts associated with dilated left ventricle, dilated cardiomyopathy, cardiomyopathy, and hypertrophic cardiomyopathy (Figure 4E,F).

**FIGURE 5:**
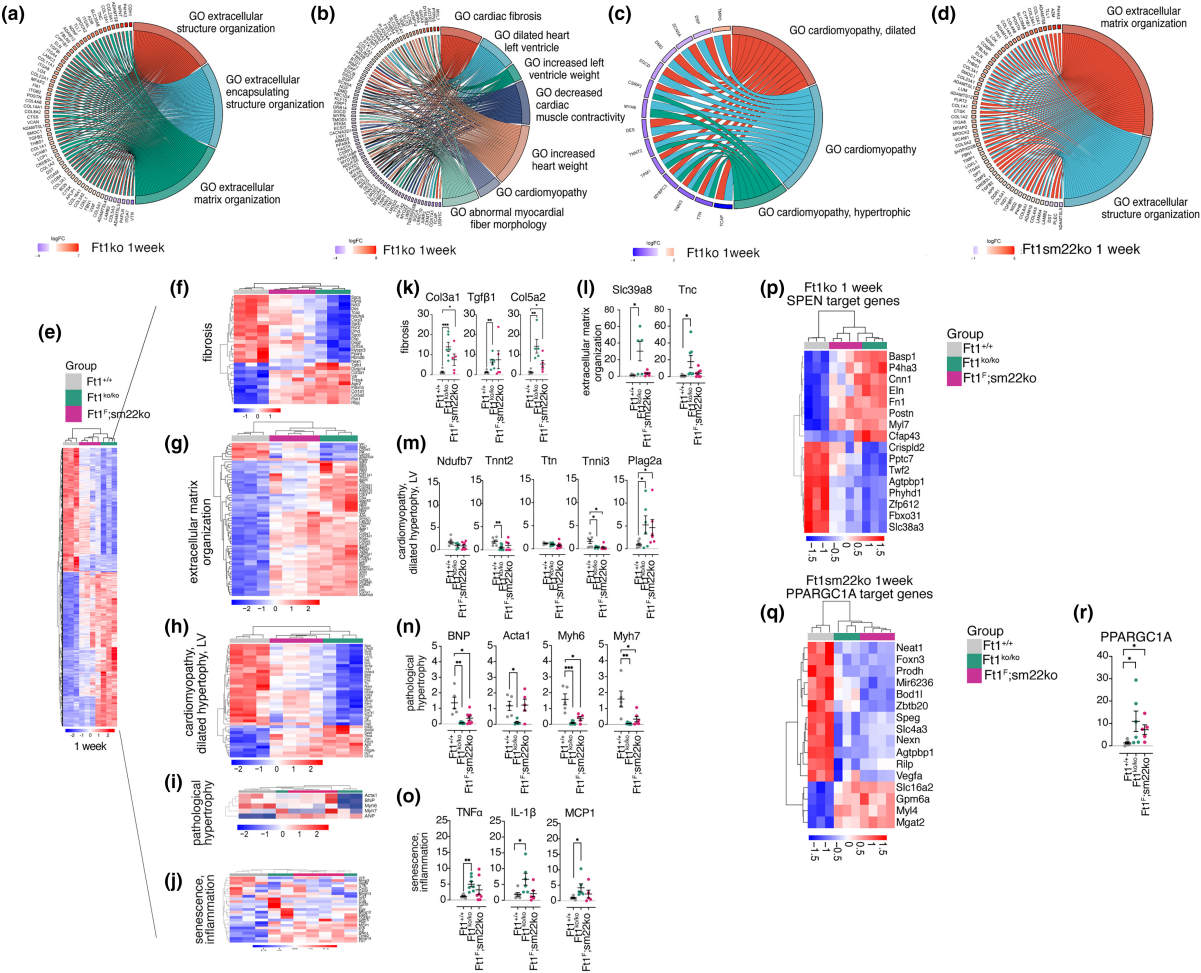
Cardiac transcriptome signatures of Ft1ko and Ft1sm22ko mice. (a–d) Chord plots showing modulated transcripts and enrichment groups from Ft1ko mice at 1 week compared to matched WT (a–c) and from Ft1sm22ko mice at 1 week compared to matched WT (d). The direction of modulation for transcript gene is indicated in the legend. Chord plots were designed on the selection from Enricher data of top terms with a *q*‐value <10^−5^. (e–j) Row‐normalized heatmaps of transcript modulation in WT, Ft1ko, and Ft1sm22ko mice at 1 week. (k–o) Q‐PCR on heart derived cDNA from WT, Ft1ko, and Ft1sm22ko mice at 1 week. (p, q) Row‐normalized heatmaps of transcript modulation of SPEN and PPARGC1A target genes of WT, Ft1ko, and Ft1sm22ko mice at 1 week. (r) Q‐PCR of relative PPARGC1A expression in the hearts from WT, Ft1ko, and Ft1sm22ko mice at 1 week. Significantly modulated transcripts were obtained by Bonferroni correction, adjusted‐*p* value <0.05. A minimum of five animals/genotype was used for Q‐PCR analyses. Q‐PCR analyses were performed on sex balanced populations. Statistical analysis was performed with Student's test and shown in graphs by *p < 0.05, **p < 0.01, ***p < 0.001. Graphs show the mean ± SEM.

To further interpret transcriptome data from the two models, we constructed chord plots on the full list of significantly modified transcripts of Ft1ko and Ft1sm22ko mice. Ft1ko mice showed upregulation of transcripts relating to the extracellular matrix, fibrosis, alteration of the left ventricle, and heart weight, cardiomyopathy (Figure 5A–C). In Ft1sm22ko mice, the alterations were mainly related to the extracellular matrix (Figure 5D).

We next built heat maps to compare WT and Ft1sm22ko to Ft1ko. The data show intra‐ and inter‐sample similarity of the Ft1ko and Ft1sm22ko signatures (Figure 5E). The analysis of single transcripts from RNAseq data related to the fibrotic signature showed upregulation of Tgfβ1, Col1a1, Col5a2, and Fbn1 in Ft1ko mice (Figure 5F). In Ft1sm22ko mice, these transcripts were less significantly modulated (Figure 5F). In the extracellular matrix group, in RNAseq data we observed the up‐regulation of a large set of collagens (Col16a1, Col24a1, Col11a1, Col12a1, Col3a1, Col14a1, Col23a1, Col5a2, Col1a2, Col5a1, Col8a2, Col1a1, Col4a6) (Figure 5G). The upregulation of members of the Adam metalloproteinase family was also detected. The hypertrophy and cardiopathic signatures of the transcriptome include prevalently downregulated transcripts and most significantly in Ft1ko mice (Figure 5H). The pathological hypertrophy signature of the transcriptome revealed that ANP is significantly upregulated most significantly in Ft1ko mice, whereas BNP, Acta1, Myh6, and Myh7, are most significantly downregulated in Ft1ko mice (Figure 5I). The senescence and inflammatory signature of the transcriptome included the modulation of TNFɑ, IL‐1β, −6, −7, MMP13, and MCP1 (Figure 5J).

Of the transcripts related to fibrosis, the expression of Col3a1, Tgfβ1, and Col5a2 in Ft1ko was assessed by Q‐PCR (Figure 5K). The modulation of Tgfβ1 is particularly relevant as this factor belongs to a family playing a central role in cardiac repair, regeneration, and ventricular remodeling by affecting cardiomyocyte growth, fibroblast activation, and extracellular matrix deposition (Berk et al., 2007; Schultz Jel et al., 2002). Of the transcripts related to extracellular matrix organization, Q‐PCR analysis proved the modulation of Slc39a8 and Tnc, a zinc transporter and an extracellular matrix component, respectively, whose dysregulation has been associated with cardiac defects (Lin et al., 2018; Shettigar et al., 2016) (Figure 5L). Of transcripts associated with cardiomyopathy, hypertrophy, and left ventricle alterations, Q‐PCR analysis was used to monitor the modulation of Ndufb7, Tnnt2, Ttn, Tnni3, and Plag2a. In all samples, we found either a significant or a tendency to downregulation in Ft1ko but not in Ft1sm22ko samples; Plag2a was upregulated in both models (Figure 5M). Of the transcripts related to hypertrophy Q‐PCR was used to monitor the modulation of BNP, Myh7, Myh6, and Acta1, whose dysregulation was associated to pathological hypertrophy (Stanley‐Hasnain et al., 2017). We found a significant decrease in their expression in Ft1ko mice. In Ft1sm22ko samples we observed a significant modulation, but for Acta1, albeit less prominent than in Ft1ko (Figure 5N). Of the transcripts related to senescence and inflammation, Q‐PCR analysis showed the modulation of TNFα, IL‐1β, and MCP1 (Figure 5O).

To identify transcription targets, we selected the 100 top modulated transcripts and interrogated the GSEA data base. We found a pivotal role for SPEN and PPARGC1A in Ft1ko and Ft1sm22ko mice, respectively (Figure 5P,Q). SPEN is a transcriptional regulator that plays a role in development, differentiation, and cancer progression (Legare et al., 2017). Interestingly, SPEN has been shown to interact with components of the Tgfβ1 signaling pathway and reported to regulate the expression of Myh6 (Rattka et al., 2021). PPARGC1A is a transcriptional coactivator regulating energy metabolism and mitochondrial function that is highly expressed in tissues with high energy demands, including the heart (Eivers et al., 2012). Q‐PCR analysis revealed the significant upregulation of PPARGC1A in Ft1ko and Ft1sm22ko (Figure 5R).

Finally, we explored by Q‐PCR the modulation of single transcripts related to pro‐fibrotic and pro‐inflammatory processes in 21 week old mice. The data show that Col3a1, Col5a2, MCP1, MMP2, iNOS are significantly upregulated in both Ft1ko and Ft1sm22ko mice. For Tgfβ1, TNFα, IL‐1β, and IL‐18 we observed their significant upregulation in Ft1ko mice and a tendency in Ft1sm22ko mice (Figure S3).

Taken together these data molecularly describe the different cardiac defects of our mutant animals, including the presence of inflammatory, senescence, fibrosis, and cardiopathy‐related factors.

### 2.4 Ft1 driven cardiac defects are rescued by p53 depletion

**FIGURE 6:**
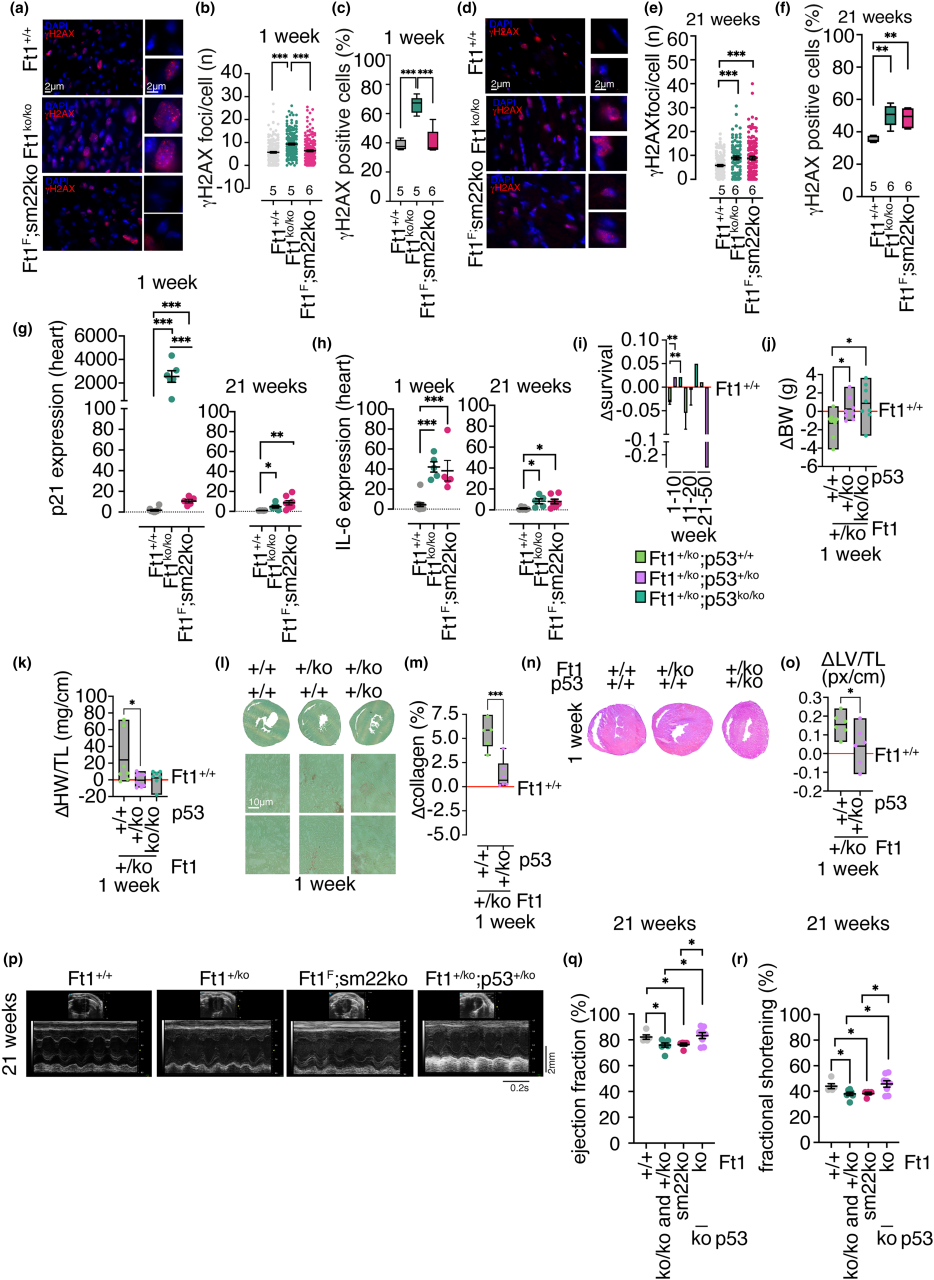
Rescue of heart defects in Ft1/p53 double mutant mice. (a–c) Representative images of DAPI (blue) and γH2AX (red) stained transverse sections of hearts from WT, Ft1ko, and Ft1sm22ko mice at 1 week and relative quantification of the number of γH2AX foci/cell and percentage of cells showing more than 5 γH2AX foci. (d–f) Representative images of DAPI (blue) and γH2AX (red) stained transverse sections of the hearts from WT, Ft1ko, and Ft1sm22ko mice at 21 weeks and relative quantification as in b and c. (g) Q‐PCR of relative p21 expression in the hearts from WT, Ft1ko, and Ft1sm22ko mice at 1 and 21 weeks. (h) Q‐PCR of relative IL‐6 expression in the hearts from WT, Ft1ko, and Ft1sm22ko mice at 1 and 21 weeks. (i) Delta survival proportion of Ft1/p53 double mutant mice vs WT. (j) Delta body weight of female Ft1/p53 double mutant vs WT at 1 week. (k) Delta HW normalized to TL of Ft1/p53 double mutant 1 week old mice vs WT at 1 week. (l) Representative images from picrosirius fast green stained transverse sections of Ft1/p53 double mutant and WT mice at 1 week. (m) Quantification of collagen percentage from images in (l) from Ft1/p53 double mutant versus WT at 1 week. (n) Representative images from hematoxylin and eosin‐stained transverse sections of the hearts of Ft1/p53 double mutant and WT mice at 1 week. (o) Quantification of delta LV thickness normalized to TL on images in (n) from Ft1/p53 double mutant mice vs WT at 1 week. (p–r) Representative images of echocardiographic tracings and analysis of ejection fraction and fractional shortening of the hearts of WT, Ft1ko/Fthz, Ft1sm22ko, and Ft1/p53 double mutant mice at 21 weeks. A minimum of five animals/genotype was used for analyses. Unless specified, analyses were performed on sex balanced populations. Statistical analysis was performed with Student's test and shown in graphs by *p < 0.05, **p < 0.01, ***p < 0.001. Graphs show the mean ± SEM.

The organismal, histological, and transcriptomic data described above show that Ft1 depletion drives heart defects, with different intensities and time profiles in Ft1ko and Ft1sm22ko mice. We next investigated the implication of DNA damage in the mouse phenotypes. The analysis of DNA damage‐associated ɣH2AX positive foci in transversal heart sections showed that Ft1ko animals display significant damage at 1 and 21 weeks (Figure 6A–F). A difference was also detected in Ft1sm22ko mice, but only at 21 weeks of age (Figure 6D–F). The level of the DNA damage biomarker p21 in cardiac tissue was robustly increased in 1 week old Ft1ko and declined at 21 weeks (Figure 6G). In Ft1sm22ko mice, there was a mild increase of p21 at 1 week, comparable to that observed at 21 weeks (Figure 6G). We next analyzed the levels of IL‐6. This inflammatory interleukin is activated by several pathways involved in DNA damage (Rodier et al., 2009; Wu et al., 2023). Q‐PCR analysis in cardiac tissue obtained from mice at 1 and 21 weeks showed a significant, acute increase of IL‐6 in at 1 week at similar levels in Ft1ko, and Ft1sm22ko animals (Figure 6H). The activation of IL‐6 declined at 21 weeks in both Ft1ko and Ft1sm22ko mice (Figure 6H).

To further explore the implication of DNA damage in the phenotype of our mice, we crossed Ft1ko mice with mice depleted of the DNA damage responder p53 (La Torre et al., 2018). As we used both homozygous and heterozygous mutant mice, we analyzed the phenotype of Ft1 heterozygous mice (Ft1hz). A partial reduction of Ft1 expression in Ft1hz mice (Figure S4A) caused defects like those in Ft1ko animals in terms of survival, body weight, heart weight, collagen volume fraction, left ventricle, blood pressure, and treadmill test (Figure S4B–J).

In double Ft1/p53 mutant mice, data on survival and body weight showed that the depletion of p53 rescued the defects of Ft1 depleted mice (Figure 6I,J). Moreover, the hearts of double mutant mice have a weight and collagen composition similar to WT mice (Figure 6K–M). Importantly, the reduction of p53 significantly rescued the alteration of the left ventricle (Figure 6N,O) and the blood pressure and physical performance defects (Figure S5A,B).

To complete the analysis of the impact of Ft1 depletion on cardiac function, we analyzed in Ft1ko, Ft1sm22ko, and double Ft1/p53 mutant mice echocardiography parameters. The results show a reduced ejection fraction and fractional shortening in both Ft1ko and Ft1sm22ko mice of 21 weeks (Figure 6P–R and Table S1). Importantly p53 depletion rescued both parameters in double Ft1/p53 mutant mice (Figure 6P–R and Table S1).

These results indicate that the constitutive depletion of Ft1 drives a robust DNA damage response that is linked with organismal and heart molecular, histological, and functional defects. On the other hand, an inflammatory process is present in both mouse models that was prominently manifested in animals at 1 week of age. Additionally, the depletion of p53, a mediator of the cell response to DNA damage, rescued the heart, and organismal defects in Ft1ko mice.

## 3 DISCUSSION

Ft1 is a factor enriched at the nuclear envelope whose depletion activates a robust DNA damage response and the accumulation of multiple telomeric signals at chromosome ends, a phenotype also described as telomere fragility (Burla et al., 2015, 2016; Cenci et al., 2015; La Torre et al., 2018, 2022). This phenotype in cells translates at the organismal level into premature aging of skin and bone and in cancer aggressiveness (Burla et al., 2015, 2016; La Torre et al., 2018). We reasoned that exploring the association of Ft1 with heart disease would expand our knowledge on Ft1 and provide insights on the pathogenic cascades and the cell types arising from DNA damage to heart defects. To investigate this latter aspect, we generated constitutively Ft1ko mice in addition to mice with vasculature targeted depletion of Ft1. The sm22 promoter is a prototypical marker of vascular smooth muscle cells (Moessler et al., 1996; Skelly et al., 2018) used because of the role of these cells in the heart. Indeed, an association with smooth muscle cells is described in the development of atherosclerotic plaques, in restenosis, and in increased extracellular matrix production (Gray et al., 2015; Hamczyk et al., 2018). Moreover, damaged smooth muscle cells may undergo hypertrophic remodeling, leading to increased vessel wall thickness and reduced vessel compliance (Gray et al., 2015). On smooth muscle cells in the heart, however, open questions remain. In particular, it is not known to what extent their alteration is the cause or consequence of heart defects, or their sensitivity to DNA damage (Wu et al., 2023).

Our data show that diffuse and targeted Ft1 depletion induce inflammation, hypertrophy, and fibrosis. The results in Ft1ko mice demonstrate, however, that the constitutive depletion of Ft1 is required to generate robust cardiac DNA damage including the activation of p21 expression (>2000 fold compared to controls), the decrease in survival and body weight, along with cardiac defects early in life. This implies that the reduction of Ft1 restricted to sm22 cells is sufficient to generate pathological heart defects, but does not induce early cardiomyopathy as does the constitutive depletion of Ft1. The differential phenotype of Ft1ko and Ft1sm22ko mice could be linked to the amount of DNA damage needed to generate cardiomyopathy, or to the type of cells that are involved. It is also possible that residual Ft1 expression can compensate for the intrinsic vascular defect, even if the phenotype of Ft1hz mice is closer to that of Ft1ko than to that of Ft1sm22ko mice. In either scenario, the reduction of Ft1 in sm22 cells creates an overall milder pathological setting compared to Ft1ko mice, especially in animals of 1 week of age.

Our data clearly implicate DNA damage as a driver of heart disease. In fact, the activation of p21 and accumulation of γH2AX foci was seen in Ft1 depleted mice. Further proof of the pivotal role of DNA damage in the cardiac phenotype of Ft1ko mice comes from double Ft1/p53 mutant mice. Indeed, the hearts of these mice do not display hypertrophy, fibrosis, and, importantly, the reduction of p53 rescues the alteration of the cardiomyopathic trait of enlargement of the left ventricle observed upon Ft1 depletion and the physical and functional heart performance. In general terms, our data point to the fact that when the equilibrium between damage and repair is lost, DNA damage and its molecular consequences drive organismal damage. When this response is attenuated, as in the absence of the DNA damage responder p53, the defects are less prominent.

A further link of our model with DNA damage and with the implication of p53 is given by the analysis of senescence associated factors in our models. Senescence is activated through two pathways: one dependent on p53 and p21 and the other on pRB and p16. RNAseq analysis showed comparable levels of p16 expression in Ft1ko and WT mice, while we observed increased expression levels of p21 in Ft1ko and Ft1sm22ko mice. This suggests that Ft1 depletion induces senescence in our models through the p53/p21 axis. Coherently with our data, a recent study in mice depleted of the DNA repair endonucleases xeroderma pigmentosum complementation group G (XPG) and excision repair cross‐complementation group 1 (ERCC1) showed that the induced dysfunction of DNA repair generated cardiac dysfunction and fibrosis, with p53 identified as a pivotal mediator of the molecular phenotype (de Boer et al., 2023). Interestingly, we observed greater activation of p21 and IL‐6 at 1 week compared to 21 weeks. A hypothetical explanation for this observation could be the exhaustion of specific factor secreting cell sources (Cowling et al., 2019) in mutant mice at 21 weeks.

Observational, epidemiological, and genetic studies in humans have highlighted the association between telomere shortening and cardiovascular disease (Martinez & Blasco, 2018). The current discussion is still controversial; however telomeric function, cell senescence, and cardiac regeneration have also been related, which prompts the speculation that these aspects could represent part of the causative elements of the cardiac phenotype observed in our mutant mice, given the telomeric role of Ft1 assessed in this and in our previous studies (Burla et al., 2015, 2016, 2018; La Torre et al., 2018; Merigliano et al., 2021).

RNAseq analysis of our mice at 1 week of age returned a clear ‐*omic* picture of the hearts that, on one hand, underlines the concept that RNAseq data bases are a reliable source of biomarkers to define heart disease, and, on the other, provides information that can be used for in silico customization and development of therapeutic strategies to combat heart disease. In fact, the RNAseq results clearly mirror organismal data. The fibrotic signature of the hearts is, for example, retrieved in the upregulation of collagen transcripts, members of the Adam metalloproteinase family and Tgfβ1, which is particularly relevant given its activity in the heart (Berk et al., 2007; Schultz Jel et al., 2002). Importantly, the phenotypic differences between Ft1ko and Ft1sm22ko mice are also underlined by transcriptomic data. These include the cardiopathic signature present in Ft1ko mice at 1 week, and the transcription control, which is SPEN‐related in Ft1ko, and PPARGC1A‐related in Ft1sm22ko mice.

A further element of interest of this study is the implication of age in the phenotype of our mice. This aspect emerges when considering several pieces of evidence. At 1 week of age, we observed an increase in nuclear numbers per area and the activation of the inflammatory cytokine IL‐6 in both Ft1ko and Ft1sm22ko mice which was not observed at 21 weeks. In this context, it appears important to stress that 1 week of age is the window during which mice display heart cell proliferation and regeneration potential (Porrello et al., 2011; Puente et al., 2014). It is tempting to speculate that this aspect drives the phenotypic traits of mutant mice. A second piece of evidence on the role of age in the phenotype of Ft1 depleted mice comes from analysis of the progression of the pathological phenotype in Ft1sm22ko mice. In this model, in fact, while we observed pre‐pathological traits in animals at 1 week, such as collagen deposition and fibrosis, we did not observe a reduction in survival or body weight early in life. However, these aspects are exacerbated and became manifest as mice age. This suggests that it is the combination of age‐induced fragility and of Ft1‐induced autocrine and, possibly, paracrine alterations to determine the phenotype of Ft1sm22ko animals. A third aspect linked with age is collagen deposition. This trait is linked with cardiac fibrosis and heart disease and is driven by pro‐fibrotic factors as Tgfβ1 (Cowling et al., 2019; Hinderer & Schenke‐Layland, 2019) that we found upregulated in our mice. Histological and molecular data show collagen accumulation in mutant mice at 1 week. At 21 weeks we observed a milder upregulation of Col3a1 and Col5a2 transcripts and no significant further augmentation of the picrosirius stained fibers. The absence of further accumulation of collagen fibers at 21 weeks could be either due to a saturation limit in fiber alteration or to the fact that the alterations of collagen that were detected via transcript analyses could not be quantitatively revealed histologically.

Taken together, our results suggest that Ft1 deficiency is a driver of DNA damage and cardiac defects. Furthermore, Ft1 deficiency targeted to vasculature smooth muscle cells is sufficient to generate a pre‐pathological profile, while its constitutive depletion, which includes its reduction in cardiomyocytes and fibroblasts in the heart, is necessary to drive manifest early and robust cardiac pathological defects. The present data add knowledge about Ft1 and the molecular cascade linking DNA damage to heart defects and contribute to the molecular information that can be exploited for the identification of biomarkers of cardiac pathology and design of new therapies for cardiac disease, the major cause of death worldwide.

## 4 MATERIALS AND METHODS

### 4.1 Mice and cells

Ft1kof animals containing the gene trap construct (La Torre et al., 2018) were crossed with flpo/Gt(ROSA)26Sorflpo mice (Birling et al., 2012). Ft1 floxed mice (Ft1F) were crossed with CMV‐cre/Tg(CMV‐cre)1Cgn (Schwenk et al., 1995) to generate Ft1ko mice. For the production of vasculature targeted Ft1 depletion, Ft1F mice were crossed with sm22‐cre/Tg(Tagln‐cre)1Zli mice (Moessler et al., 1996). To obtain double mutants, Ft1ko mice were crossed with p53+/− animals (La Torre et al., 2018). All animals were kept in standard laboratory conditions (temperature 21 ± 2°C, humidity 60 ± 10%, according to a 12 h light dark cycle). Standard mouse cages were used, all animals were kept with environmental enrichments (mice igloos) and fed with food and water ad libitum. WT, Ft1ko, and Ft1sm22ko animals were weighed once a week. Offspring were weaned at 3 weeks, tail biopsies were used to genotype animals, and Ft1 expression was analyzed by Q‐PCR in sex balanced populations. Body weight analysis was performed on female mice. Survival curve was obtained from sex balanced populations. When needed, mice were euthanized by asphyxiation with carbon dioxide or cervical dislocation. All procedures were performed under the approved protocol 693/2022‐PR (7FF2C.15). Mouse embryonic fibroblasts were isolated and cultured as described (La Torre et al., 2018). For LV‐cre transduction of mouse embryonic fibroblasts, we used LV‐cre vector and LV‐ctr vector as previously described (Burla et al., 2015; Piersanti et al., 2015).

### 4.2 Functional analysis

Mean, systolic, and diastolic blood pressure of conscious mice was measured with the tail‐cuff method (Blood pressure analysis system, BP‐2000 series 2, Visitech system). Treadmill test was performed with treadmill control LE8710 (Panlab Instruments) at 15 degrees downhill. Before the test, mice were warmed up at 5 m/min for 5 min. For the test, the following running timing set‐up was used: 5 m/min for 2 min, 7 m/min for 2 min, 8 m/min for 2 min, and 10 m/min for 5 min. Speed was then increased 1 m/min to a final speed of 20 m/min. The inability of the animal to remain on the treadmill despite electrical prodding was used as the endpoint of the experiment—exhaustion. Analyses were performed on sex balanced populations. For echocardiographic analyses we used a VEVO 3100 (Visualsonics®—mx550d probe). Mice were anesthetized with 2.5% avertin (12 μL/g body weight, intraperitoneally). Analyses were performed on sex mixed populations.

### 4.3 Genotyping

Genomic DNA (gDNA) was extracted from tail biopsies using the NucleoSpin Tissue Extraction Kit (Macherey‐Nagel) following the manufacturer's instructions. PCR reaction was performed with Platinum Blue PCR SuperMix (Invitrogen). PCR products were resolved by electrophoresis on 1.8% agarose gels (Seakem, Lonza) containing SYBR Safe DNA Gel Stain (Merck, Life Technologies).

### 4.4 Q‐PCR

Total RNA was extracted from tissues of 1 or 21 week old mice from sex balanced populations and stored in dry ice. RNA was extracted using the TRIzol reagent (Invitrogen) following the manufacturer's instructions. RNA was reverse transcribed into cDNA with oligo d(T) primers and OMNISCRIPT RT KIT (Qiagen). Q‐PCR was performed as previously described (Remoli et al., 2015) with SYBR Green Mix (QuantiTect SYBR Green PCR Kit, Qiagen) and using QuantStudio 3 (Applied Biosystem).

### 4.5 Immunostaining and fluorescence in situ hybridization

For immunostaining, cells were fixed with 3.7% formaldehyde for 10 min at 4°C and permeabilized with 0.25% Triton X‐100 in PBS for 5 min. When needed, cells were pre‐permeabilized as previously described (Burla et al., 2015). Cells were then incubated with the following antibodies with 3% BSA: anti‐ɣH2AX (Upstate Biotechnology) and with secondary antibody anti‐rabbit‐ALEXA 555 (Invitrogen). Nuclei were visualized using DAPI (4,6 diamidino‐2‐phenylindole) and coverslips were mounted in Vectashield H‐1000.

Telomeric fluorescence in situ hybridization (FISH) was performed as previously described (Burla et al., 2015). Images were acquired with Zeiss Axioplan epifluorescence microscope equipped with a CCD camera (CoolSnap HQ; Photometrics). Images were pseudo‐colored and combined in Adobe Photoshop CC to create merged images and processed using ImageJ (http://imagej.nih.gov) and Adobe Photoshop CC.

### 4.6 Histology

Cardiac tissues were collected from 1 or 21 week old mice from sex balanced populations, weighed, and included in OCT with isopentane (Sigma) cooled in liquid nitrogen. Tissues were cryostat dissected and subjected to subsequent analyses. For morphometric analyses, cardiac sections (15 μm) were stained with hematoxylin (Carlo Erba) and eosin (Sigma) or with green picrosirius (Sigma) and mounted with DPX for Histology (Sigma). For immunohistochemical analysis, cardiac sections (9 μm) were treated as following. Briefly, sections were fixed in cold acetone for 5 min and washed twice with PBS. The nonspecific sites were blocked with a solution of PBS 0.2% Triton (Invitrogen), 0.2% BSA (Sigma), and 5% goat serum (Sigma) for 1 h at room temperature. The sections were then incubated with anti‐dystrophin antibody (Abcam) or anti‐ƔH2AX (Upstate Biotechnology) overnight in PBS and 2% goat serum at 4°C. Sections were washed with PBS and incubated for 1 h at room temperature with the secondary antibody (anti‐rabbit‐ALEXA 555, Invitrogen) and mounted with Vectashield Antifade mounting with DAPI H‐2000 for microscopic evaluation. Hematoxylin and eosin staining was performed following the manufacturer's instructions (Carlo Erba, Sigma); dehydrated sections in 95% ethanol, 100% ethanol, and xylene twice for 5 min were then mounted with DPX (Sigma). Green picrosirius staining was performed following the manufacturer's instructions (Sigma). Briefly, cardiac sections (15 μm) were incubated in 0.1% Fast Green (Thermo Fisher Scientific) for 60 min. Sections were then washed with tap water and incubated in 1% acetic acid for 1 min. Sections were washed with distilled H_2_O and incubated in 0.1% of Sirius Red for 2 min and then washed with distilled H_2_O. Afterwards, sections were dehydrated in 95% ethanol, 100% ethanol, and xylene for 5 min twice and then mounted with DPX (Sigma). Images were taken with the ZEISS‐Axio Phot microscope (Zeiss) connected to the Progress‐C5 JENO‐PTIK camera with PROGRESS MAC (Capture PRO) software.

### 4.7 Morphometric analyses

Morphometric analyses of cardiac ventricle walls were performed on sections stained with either hematoxylin and eosin or green picrosirius. The thickness of the walls was analyzed with ImageJ. Morphometric analysis of CSA was performed on dystrophin‐stained sections with ImageJ. Nuclei/field analysis was performed on DAPI stained sections with ImageJ. Percentage of collagen was measured on stained cardiac sections with ImageJ as previously described (Han et al., 2023).

### 4.8 RNAseq

Total RNA was extracted from cardiac tissue of male 1 week old mice (3 WT, 3 Ft1ko, 4 Ft1sm22ko animals/group). RNA was extracted using the TRIzol reagent (Invitrogen) following the manufacturer's instructions. RNAseq was performed by IGATech. Universal Plus mRNA‐Seq kit (Tecan Genomics, Redwood City, CA) was used for library preparation following the manufacturer's instructions (library type: fr‐secondstrand). RNA samples were quantified, and quality was tested with the Agilent 2100 Bioanalyzer RNA assay (Agilent technologies, Santa Clara, CA). Final libraries were checked with both Qubit 2.0 Fluorometer (Invitrogen, Carlsbad, CA) and Agilent Bioanalyzer DNA assay or by Caliper LabChip GX (PerkinElmer, Waltham, MA). Libraries were then prepared for sequencing and sequenced on paired‐end 150 bp mode on NovaSeq 6000 (Illumina, San Diego, CA). RNAseq reads were analyzed via Illumina pipeline and then aligned on reference hg38 genome/transcriptome with STAR4 (default parameters). Transcript count was done with Stringtie using default parameters. RSeqQC6 package was used for quality control. Pair‐wise differential expression analysis was performed as follows. Htseq‐count7 was used to preprocess RNAseq data for differential expression analysis by counting the overlap of reads with genes. DESeq28,9 was then used to perform comparisons between expression levels of genes and transcripts by fitting a Generalized Linear Model (GLM) for each gene as previously described (Anders & Huber, 2010). The list of transcripts passing the Bonferroni correction was used to build volcano plots, Venn diagrams, and then uploaded into the Enrichr analysis tool for further interpretation (Xie et al., 2021). To construct chord plots, we exported Enrichr data via Appyter, selected top terms with a *q*‐value equal or below 10^−5^ and exported the data to SRPlot (https://www.bioinformatics.com.cn/en). Row‐normalized heatmaps were built with SRPlot. GSEA (http://software.broadinstitute.org/sea/) was used to define transcription control on the 100 top modulated transcripts. The RNAseq data discussed herein are deposited in NCBI's Gene Expression Omnibus (Edgar et al., 2002) and are accessible through GEO Series accession number GSE236989 (https://www.ncbi.nlm.nih.gov/geo/query/acc.cgi?acc=GSE236989).

### 4.9 Statistics

Kaplan–Meier curves were analyzed in sex balanced populations using the log‐rank (Mantel‐Cox) test. Independent datasets were compared with Student's *t*‐test (unpaired, two‐tailed). p < 0.05 was used as cutoff to determine statistically significance and the following parameters were used to show statistically significant differences in graphs * p < 0.05, ** p < 0.01, *** p < 0.001 in Student's *t*‐test. Unless specified, graphs show the mean ± SEM.

## AUTHOR CONTRIBUTIONS

Mattia La Torre, Eleonora Centofante, Carmine Nicoletti, Romina Burla, Alessandro Giampietro, Federica Cannistrà, Leonardo Schirone, Valentina Valenti performed the experiments. Antonio Musarò and Sebastiano Sciarretta contributed to the design of the experiments of the manuscript and data discussion. Isabella Saggio designed the experiments and wrote the manuscript.

## CONFLICT OF INTEREST STATEMENT

The authors declare no competing interests.

## Supporting information


Figure S1.

Figure S2.

Figure S3.

Figure S4.

Figure S5.

Table S1.

Table S2.

## Data Availability

The data and materials that support the findings of this study are available from the corresponding author upon reasonable request.
