# Supplementary figures and images for: Impact of diffused versus vasculature targeted DNA damage on the heart of mice depleted of telomeric factor Ft1
Source: Aging Cell. 2023 Nov 13;22(12):e14022. doi: 10.1111/acel.14022 (PMC10726857; doi:10.1111/acel.14022)

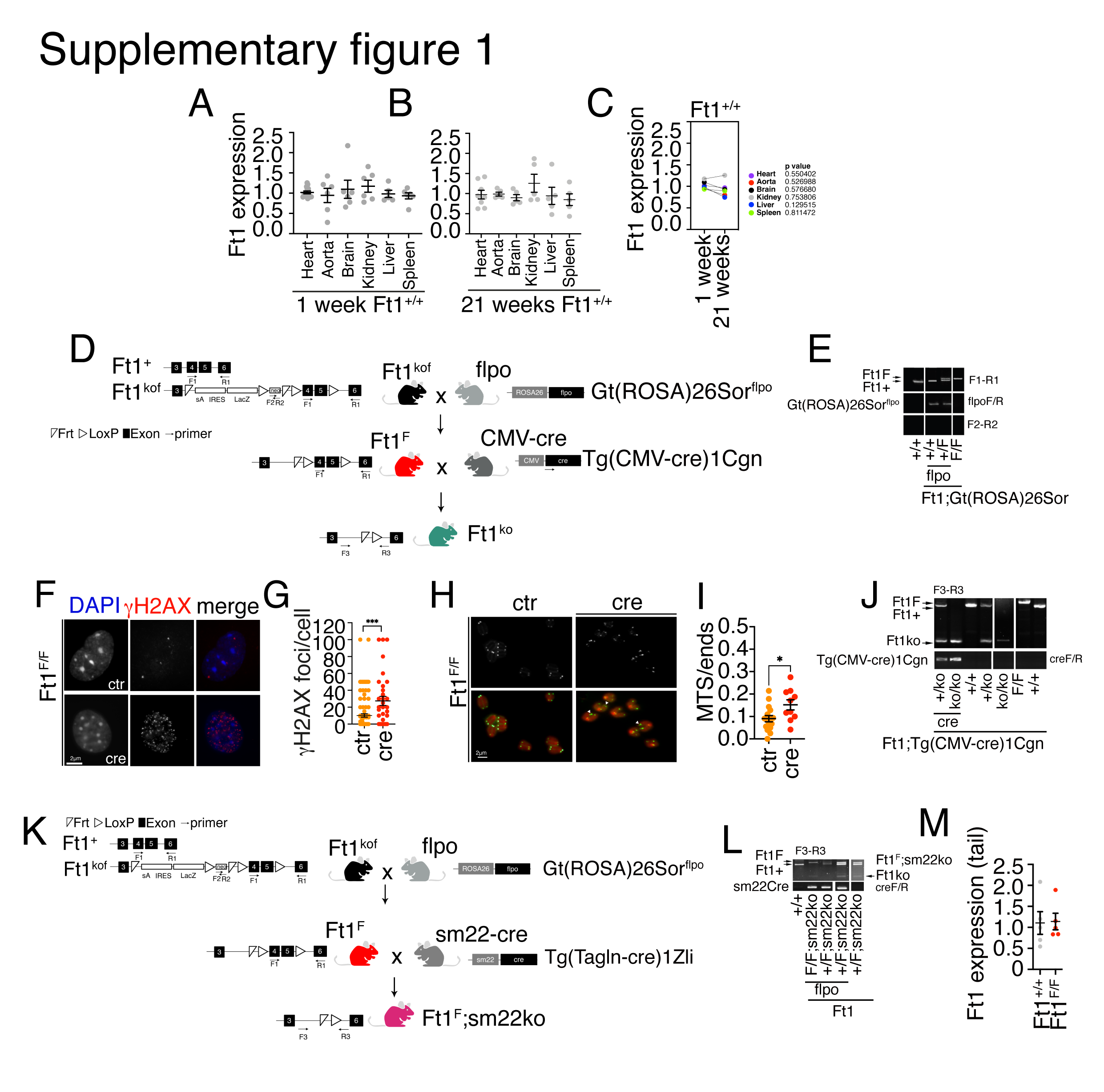

Supplement: Supplementary file 1 — Figure S1. [file ACEL-22-e14022-s004.tif]

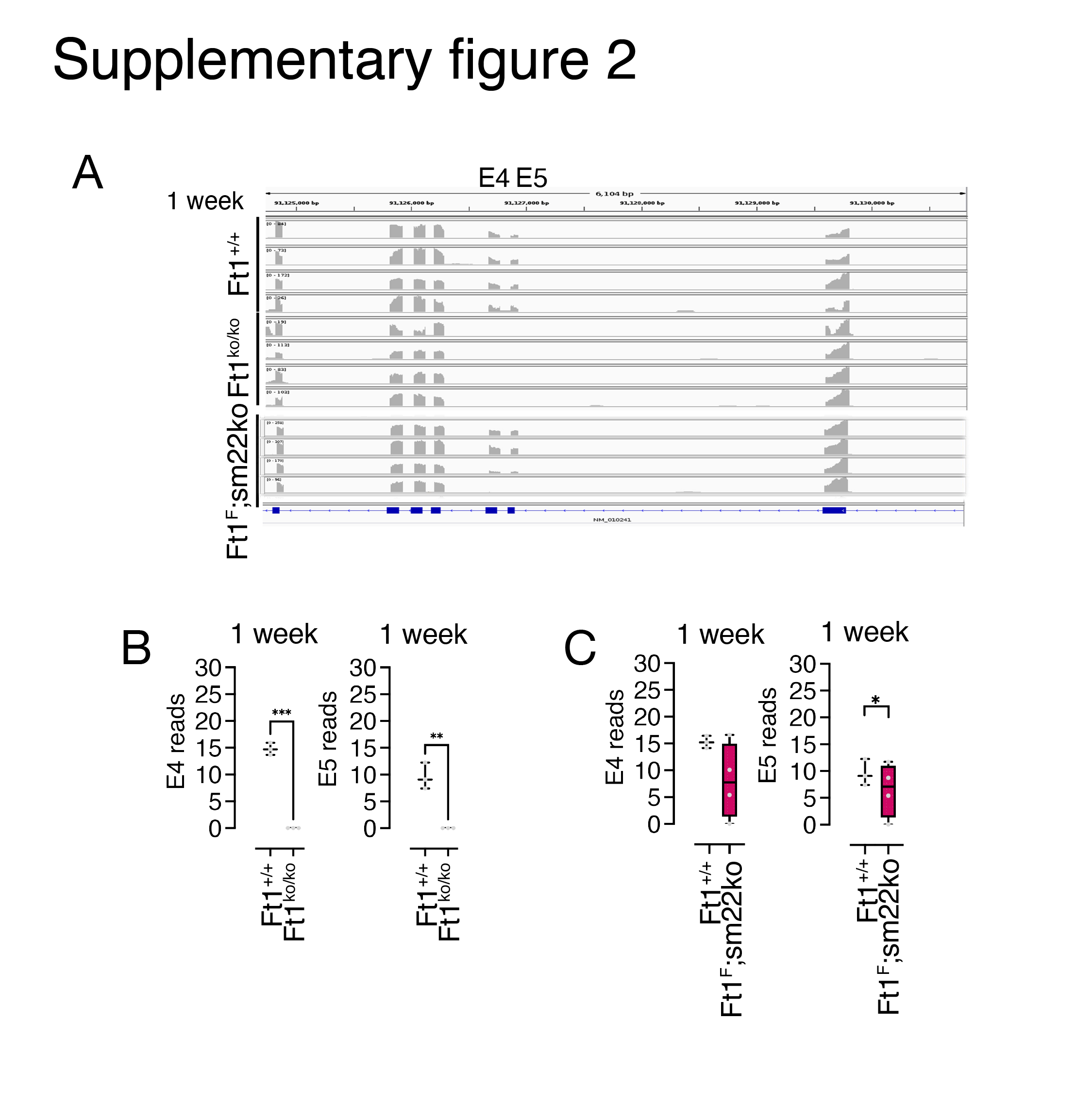

Supplement: Supplementary file 2 — Figure S2. [file ACEL-22-e14022-s007.tif]

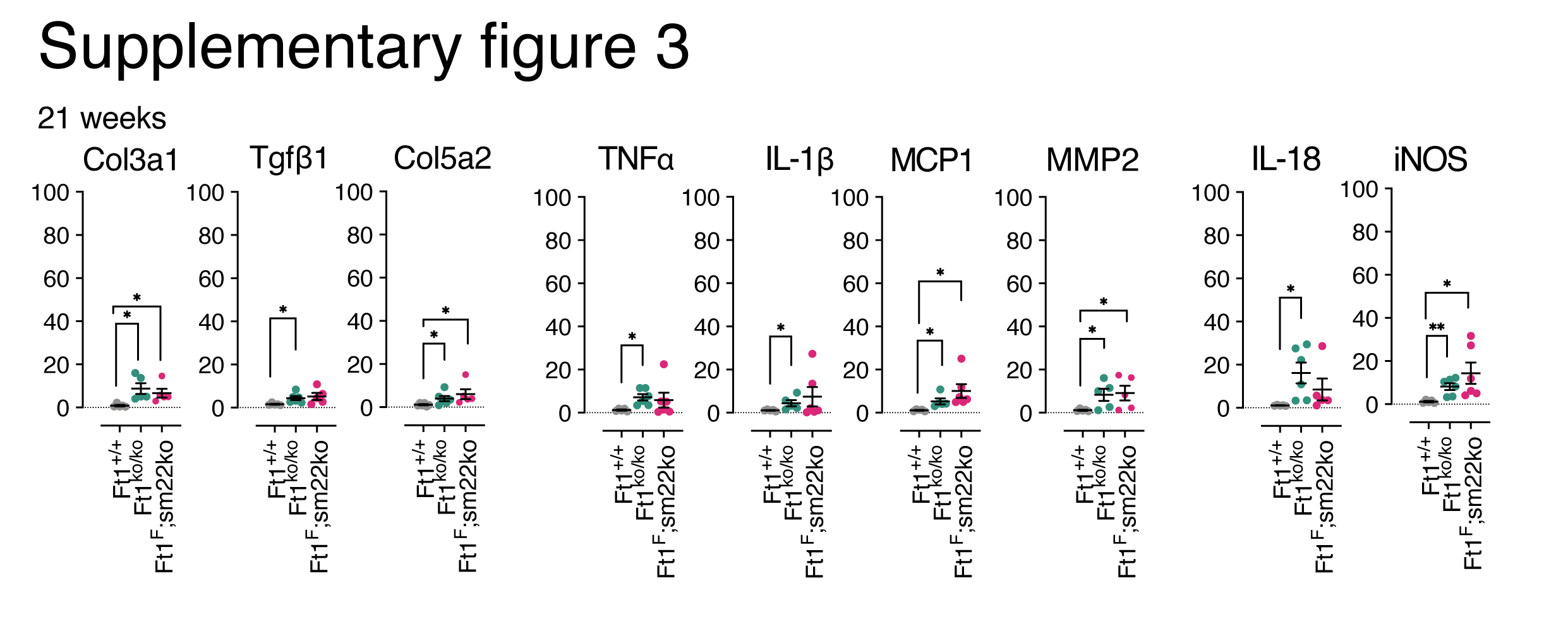

Supplement: Supplementary file 3 — Figure S3. [file ACEL-22-e14022-s008.tif]

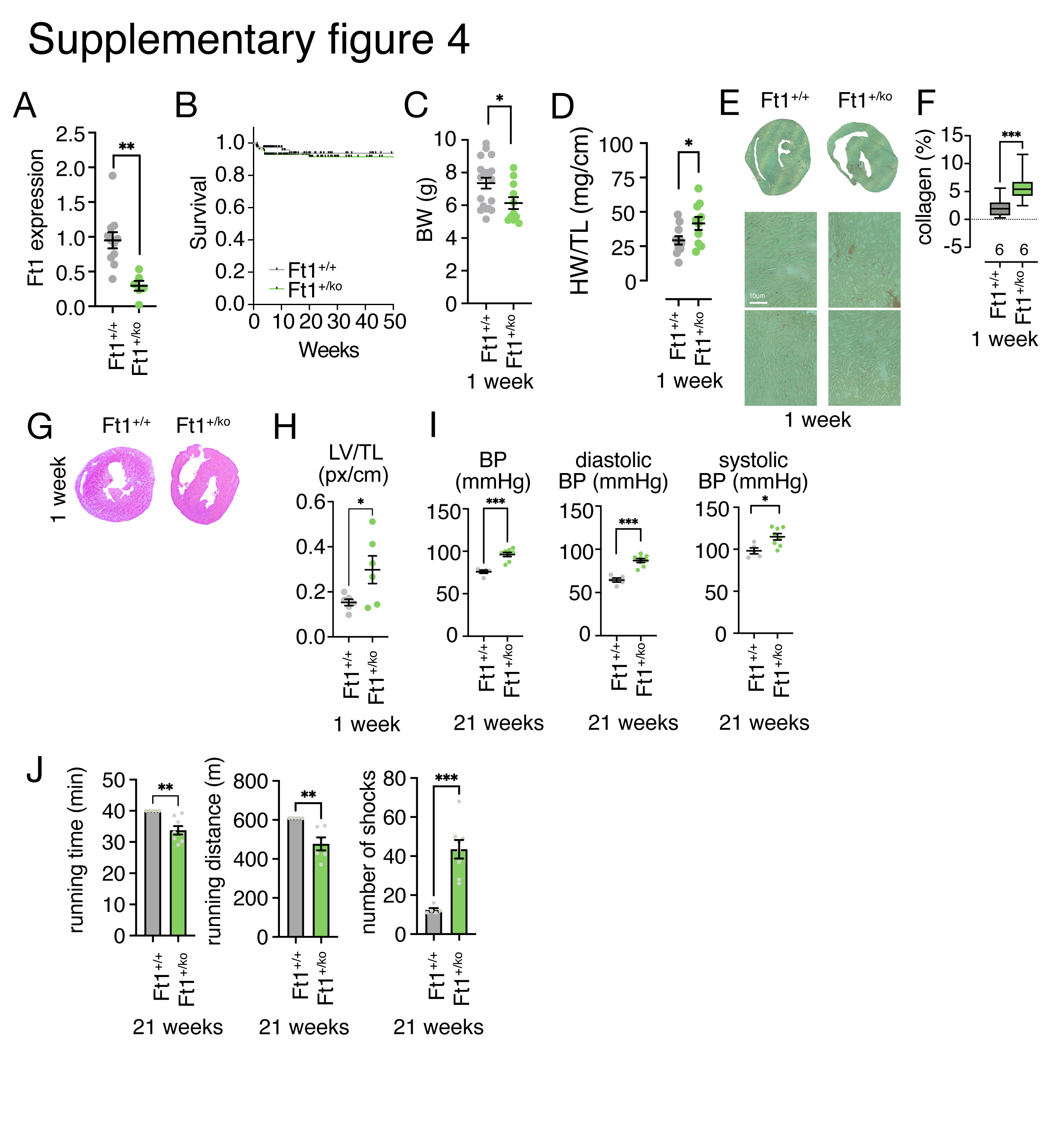

Supplement: Supplementary file 4 — Figure S4. [file ACEL-22-e14022-s002.tif]

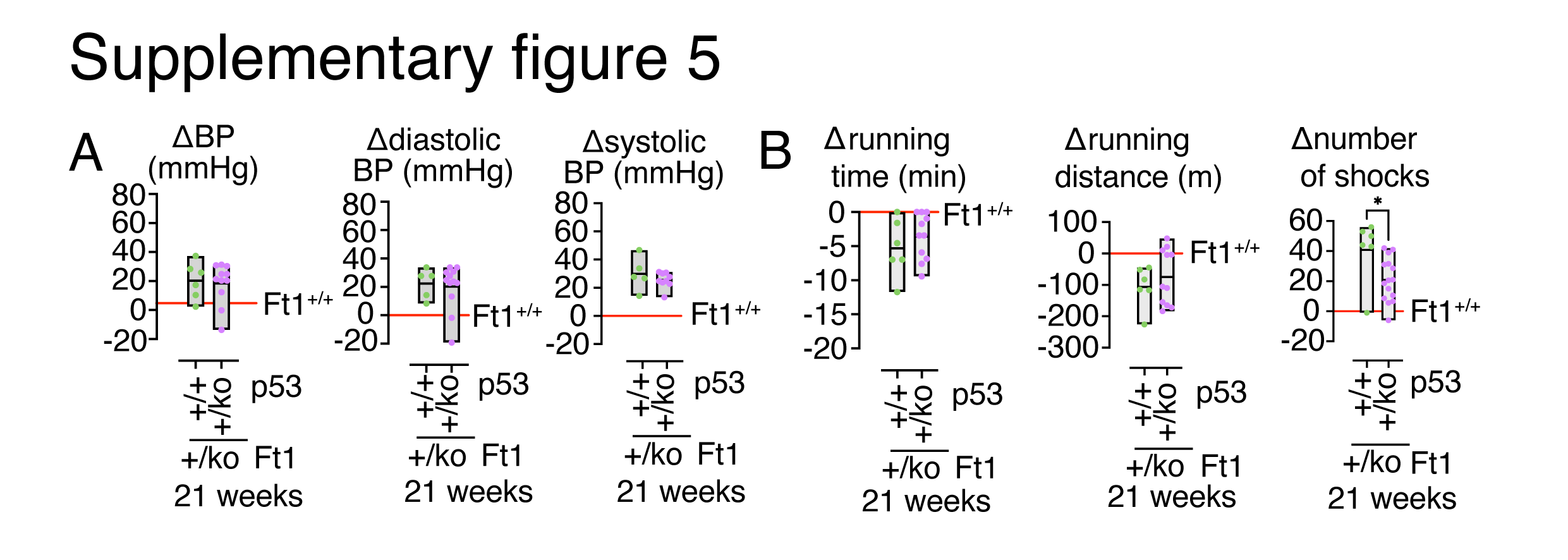

Supplement: Supplementary file 5 — Figure S5. [file ACEL-22-e14022-s003.tif]

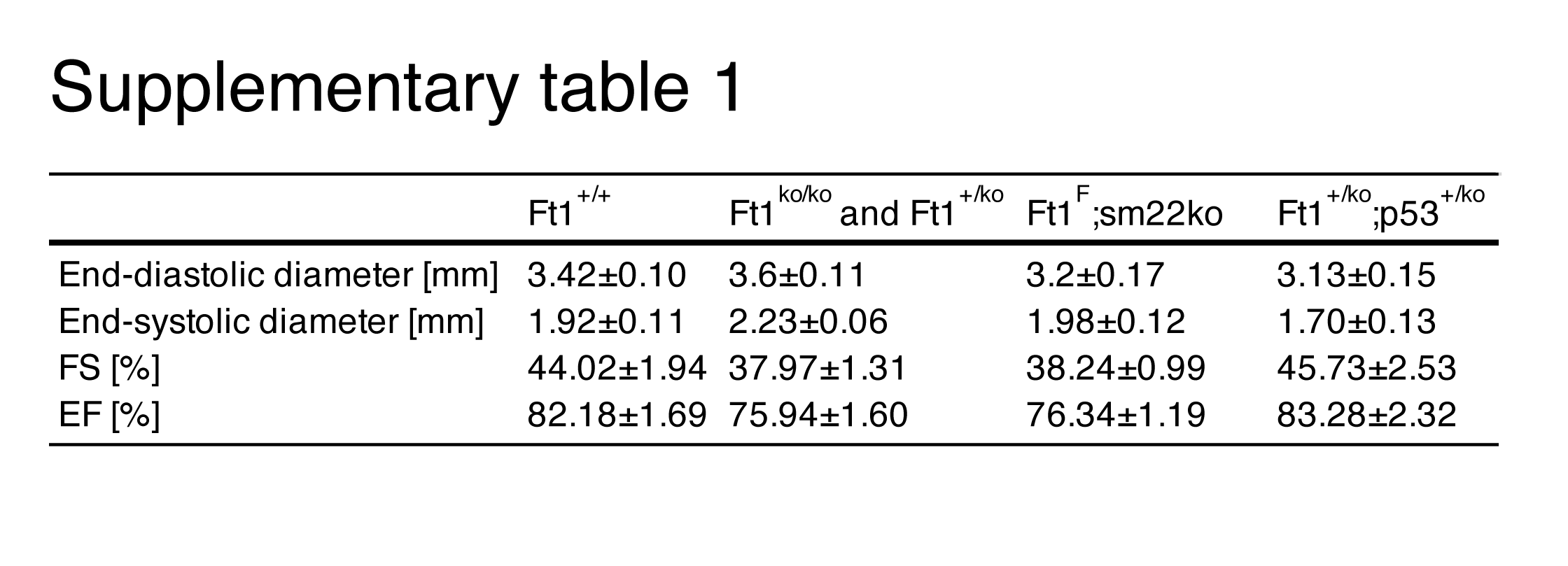

Supplement: Supplementary file 6 — Table S1. [file ACEL-22-e14022-s006.tif]

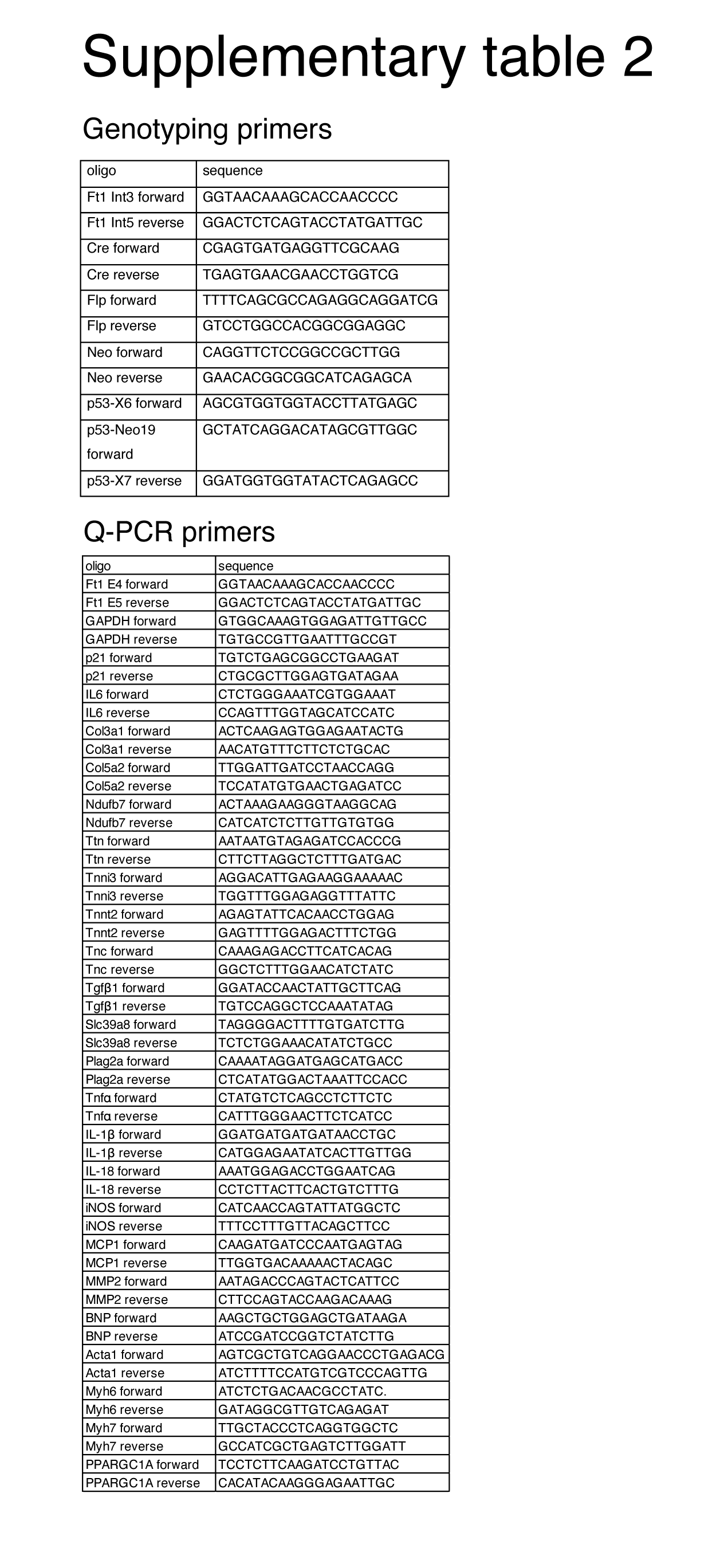

Supplement: Supplementary file 7 — Table S2. [file ACEL-22-e14022-s001.tif]
