# Supplementary material for: Impact of diffused versus vasculature targeted DNA damage on the heart of mice depleted of telomeric factor Ft1
Source: Aging Cell. 2023 Nov 13;22(12):e14022. doi: 10.1111/acel.14022 (PMC10726857; doi:10.1111/acel.14022)
Supplement: Supplementary file 8 [file ACEL-22-e14022-s005.docx]

**Supporting figure legends**

**Figure S1. Production of constitutively and vascular smooth muscle targeted Ft1 ko mice.** (A-B) Q-PCR analysis of relative Ft1 gene expression from different tissues from mice at 1 and 21 weeks. (C) Correlation analysis of Ft1 expression and mice age in different organs. (D) Schematic representation of the strategy used to generate Ft1F and Ft1ko mice. (E) Representative images of PCR genotyping of Ft1F mice. (F-G) Representative images of DAPI (blue) and ɣH2AX (red) stained LV-Cre or LV-ctr transduced Ft1F mouse embryonic fibroblasts and relative quantification. (H-I) Representative partial DAPI-stained (red) metaphases from LV-Cre or LV-ctr transduced Ft1F mouse embryonic fibroblast showing telomeric signals (black and white; green in merges) and relative quantification. (J) Representative images of PCR genotyping of Ft1ko mice. (K) Schematic representation of the strategy used to generate Ft1sm22ko mice. (L) Representative images of PCR genotyping of Ft1sm22ko mice. (M) Q-PCR analysis of relative Ft1 expression in WT and Ft1F mice. A minimum of 5 animals/genotype was used for analyses. Analyses were performed on sex balanced populations. Statistical analysis was performed with Student’s test and shown in graphs by *p<.05, **p<.01, ***p<.001. Graphs show the mean ± SEM.

**Figure S2. RNAseq analysis of Ft1 transcript alignment.** (A) Transcript reads alignment on Ft1 gene region from exons 3 to 9. (B-C) Quantification of transcript peaks on exons 4 and 5 in male WT (N=3), Ft1ko (N=3), and Ft1sm22ko (N=4) animals at 1 week. Statistical analysis was performed with Student’s test and shown in graphs by *p<.05, **p<.01, ***p<.001. Graphs show the mean ± SEM.

**Figure S3. Q-PCR analysis of Ft1 depleted 21 week old mice.** Q-PCR of relative expression of the indicated genes in the hearts from WT, Ft1ko, and Ft1sm22ko mice at 21 weeks. A minimum of 5 animals/genotype was used for analyses. Analyses were performed on sex balanced populations. Statistical analysis was performed with Student’s test and shown in graphs by *p<.05, **p<.01, ***p<.001. Graphs show mean ± SEM.

**Figure S4. Ft1 heterozygous mice show defects like those of Ft1ko animals.** (A) Q-PCR quantification of cardiac Ft1 expression of sex balanced populations of WT and Ft1hz mice. (B) Kaplan–Meier survival curve from 1 to 50 weeks of sex balanced populations of WT and Ft1hz mice. (C) Body weight of female WT and Ft1hz mice. (D) HW normalized to TL of WT and Ft1hz mice at 1 week. (E-F) Representative images and quantification of collagen percentage from green picrosirius stained transverse sections of WT and Ft1hz at 1 week. (G-H) Hematoxylin and eosin staining and morphometric analyses of LV walls normalized to TL of WT and Ft1hz mice. (I) Mean, diastolic and systolic BP of WT and Ft1hz at 21 weeks. (J) Running time, distance and number of shocks observed in WT and Ft1hz mice at 21 weeks during an exhaustion treadmill test. A minimum of 5 animals/genotype was used for analyses. Unless specified, analyses were performed on sex balanced populations. Statistical analysis was performed with Student’s test and shown in graphs by *p<.05, **p<.01, ***p<.001. Graphs show the mean ± SEM.

**Figure S5.** **Rescue of heart pressure and run performance defects in Ft1/p53 double mutant mice.** (A) Delta mean, diastolic and systolic BP measurements of Ft1/p53 double mutant mice vs WT at 21 weeks. (B) Delta running time, distance and number of shocks observed in Ft1/p53 double mutant mice vs WT at 21 weeks observed during an exhaustion treadmill test. A minimum of 5 animals/genotype was used for analyses. Analyses were performed on sex balanced populations. Statistical analysis was performed with Student’s test and shown in graphs by *p<.05, **p<.01, ***p<.001. Graphs show mean ± SEM.

**Table S1. Table of the echocardiographic measurements.** A minimum of 5 animals/genotype was used for analyses. Analyses were performed on mixed sex populations.

**Table S2. Table of oligo used for genotyping and Q-PCR analysis.**
